# Prioritizing options for multi-objective agricultural development through the Positive Deviance approach

**DOI:** 10.1371/journal.pone.0212926

**Published:** 2019-02-25

**Authors:** Jonathan Steinke, Majuto Gaspar Mgimiloko, Frieder Graef, James Hammond, Mark T. van Wijk, Jacob van Etten

**Affiliations:** 1 Bioversity International, CGIAR Research Program on Roots, Tubers and Bananas, Turrialba, Costa Rica; 2 Horticultural Economics, Humboldt University Berlin, Berlin, Germany; 3 Sustainable Land Use in Developing Countries, Leibniz-Centre for Agricultural Landscape Research (ZALF), Müncheberg, Germany; 4 Tanzania Agricultural Research Institute, Naliendele, Tanzania; 5 Livestock Systems and the Environment, International Livestock Research Institute, Nairobi, Kenya; Kansas State University, UNITED STATES

## Abstract

Agricultural development must integrate multiple objectives at the same time, including food security, income, and environmental sustainability. To help achieve these objectives, development practitioners need to prioritize concrete livelihood practices to promote to rural households. But trade-offs between objectives can lead to dilemmas in selecting practices. In addition, heterogeneity among farming households requires targeting different strategies to different types of households. Existing diversity of household resources and activities, however, may also bear solutions. We explored a new, empirical research method that identifies promising options for multi-objective development by focusing on existing cases of strong multi-dimensional household performance. The “Positive Deviance” approach signifies identifying locally viable livelihood practices from diverse households that achieve stronger performance than comparable households in the same area. These practices are promising for other local households in comparable resource contexts. The approach has been used in other domains, such as child nutrition, but has not yet been fully implemented for agricultural development with a focus on the simultaneous achievement of multiple objectives. To test our adapted version of the Positive Deviance approach, we used a quantitative survey of over 500 rural households in South-Eastern Tanzania. We identified 54 households with outstanding relative performance regarding five key development dimensions (food security, income, nutrition, environmental sustainability, and social equity). We found that, compared to other households with similar resource levels, these “positive deviants” performed strongest for food security, but only slightly better for social equity. We then re-visited a diverse sub-sample for qualitative interviews, and identified 14 uncommon, “deviant” practices that plausibly contributed to the households’ superior outcomes. We illustrate how these practices can inform specific recommendations of practices for other local households in comparable resource contexts. The study demonstrates how, with the Positive Deviance approach, empirical observations of individual, outstanding households can inform discussions about locally viable agricultural development solutions in diverse household context.

## Introduction

In recent years, agricultural researchers and policy-makers have increasingly moved away from strategies that focus on a single goal, such as productivity or household income. Modern development paradigms, such as Sustainable Intensification [1,2] or Climate-Smart Agriculture [3] emphasize that agricultural development should pursue multiple goals at the same time, including food security, nutrition quality, and improved gender relationships. These multi-objective paradigms outline broad goals, but do not predefine interventions, though they are commonly associated with diverse practices such as agroforestry, organic farming, and farm diversification [4–6]. Choosing suitable farm-level intervention options is challenging because different contexts require different recommendations. Furthermore, trade-offs can exist between different objectives, causing dilemmas between multiple household goals [7].

To inform decision-making and design intervention strategies, various methods exist. Quantitative analysis of household data can be used for predicting the outcomes of technological and institutional change on small farms [8]. More systemic analysis considers interactions between household activities as well as trade-offs between development goals in quantitative models [9,10]. But strong complexity and systemic and behavioral uncertainties can affect the practical value of quantitative analysis for generating household-level recommendations [11]. Complementing quantitative approaches with participatory research may help to cut through this complexity and link the analysis with reality on the ground [12]. For example, to reduce the number of options to test, research has frequently subjected “best-bet” solutions to ex-ante assessments by farmers [13,14]. Participatory methods can account for context-specific considerations and preferences, but can be prone to various forms of bias, e.g., relating to the sampling of research participants [15], enumerator identity [16] or participants’ resistance to modify pre-held opinions [17].

Research approaches that combine the strengths of quantitative systems analysis and participatory research to prioritize interventions are promising as they provide complementary perspectives. Existing combined approaches, however, risk underemphasizing the heterogeneity of households [11,18]. As the adoption potential of different practices can vary strongly between households, informed targeting of practices to suitable end users is required [19,20].

A combination of quantitative and qualitative methods with explicit emphasis on household heterogeneity is the *Positive Deviance approach*. This research approach was pioneered by nutritionists to identify child nutrition improvement practices that are locally viable and acceptable [21,22]. They used quantitative survey data to identify households with exceptionally good child health indicator scores compared to other households in similar circumstances. Through follow-up visits to these “positive deviants”, the researchers identified feeding and hygiene practices unique to these households that possibly explained their superior performance. The identified practices were then promoted to other, worse-performing households in similar cultural and resource contexts [23]. In the field of agriculture, positive deviants have been playing key roles in innovation processes [24–26], and agricultural research has recently begun exploring systematic methods of identifying and learning from such outstanding farming households [27]. The Positive Deviance approach is an interesting data-driven approach that cuts through analytical complexity to provide suggestions on viable interventions, based on empirical, qualitative insights. Existing studies, however, did not explore smallholder household performance as a multi-dimensional phenomenon, and have not yet gone from identifying exceptionally well-performing households to identifying potentially superior practices. Our goal was to explore how the Positive Deviance approach can be adapted to identify and prioritize rural development interventions for diverse farming households that pursue multiple objectives. We describe the adapted approach, consisting of three research steps, and a case study implementation in Tanzania. Based on this experience, we discuss the potential of the Positive Deviance approach for household-specific prioritization of multi-objective development opportunities.

## Methods

### Overview of the approach

Step 1: indicators the first, quantitative research step, we collected household-level data that characterize farming systems and allow quantifying livelihood performance indicators. We used these data to identify positive deviant households that optimize household performance across multiple development objectives.
Step 2: In this qualitative research step, we explored positive deviants’ behaviors through interviews and farm visits, to identify uncommon practices embedded in local context. Since alternative farming styles, involving different responses to the same trade-offs, can lead households to achieve diverse, but equally optimized farm designs [28], we expected positive deviants to employ a diverse range of practices.
Step 3: Lastly, we focused on positive deviants as success cases that can be models for other households with similar resource levels. We linked the observed practices back to the quantitative data on household context to estimate which practices are likely viable solutions for which particular households. We explore the feasibility of our novel method for assisting decision-making in strategic planning of development interventions, as well as providing inputs to heuristic prioritization of viable intervention options at the household level.

### Research area

**Fig 1:**
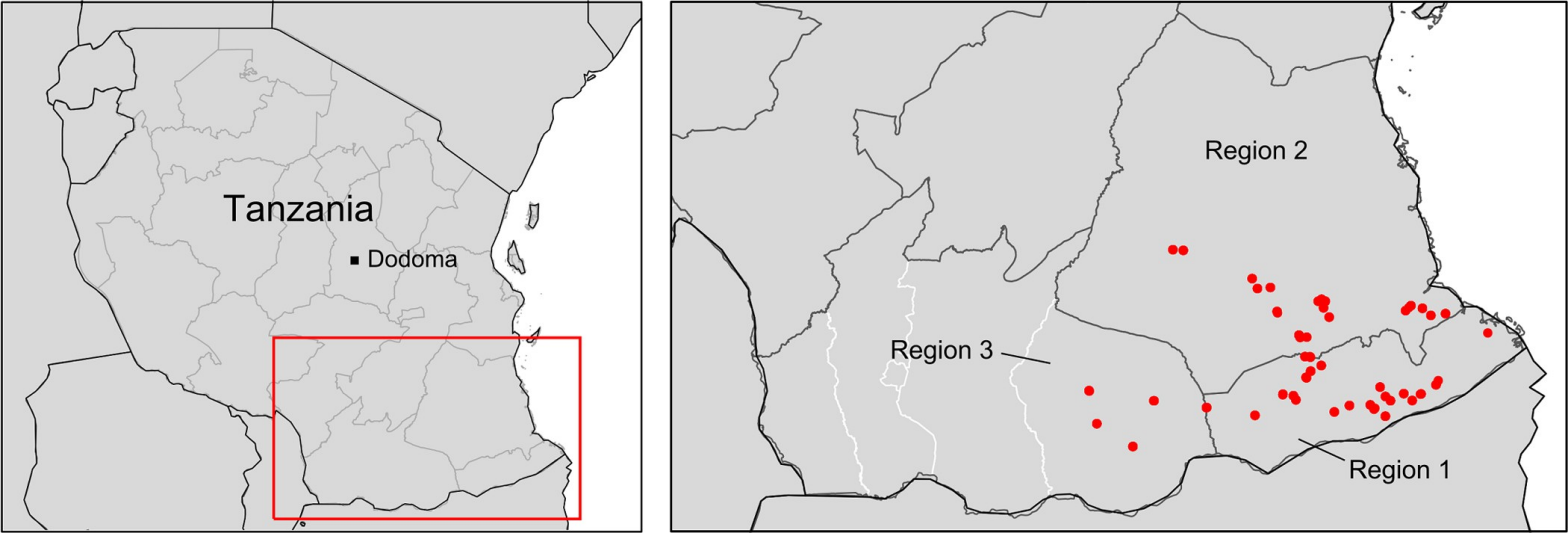
Research area. Household sampling sites are marked in red. Sub-regional district borders shown only where needed. Spatial data retrieved from gadm.org.

We conducted research in the *Southern Agricultural Zone* of Tanzania, which includes Mtwara region (Region 1, Fig 1), Lindi region (Region 2), and the Tunduru district of Ruvuma region (Region 3). Farming systems are dominated by rain-fed low-input cropping of cereals (maize, sorghum), cassava, and pulses (pigeon pea, green grams) as well as chicken husbandry for subsistence, and commercial production of pulses and oil seeds (e.g., cashew nut, groundnut, sesame). Rural population density is low (~1–5 persons/km^2^), infrastructural development has been lagging behind the national standard in recent years, and poverty rates are among the highest at national scale [29].

### Identification of positive deviants

#### Lean data household survey

We collected household data using the standardized *Rural Household Multiple Indicator Survey* (RHoMIS) [30] and calculated a set of livelihood indicators for each household (Table 1). RHoMIS provides quantitative information about individual households, including key performance variables, such as food security status and income level. It also collects data about household resources (e.g., land holdings) and the agricultural system (e.g., market orientation). To ensure data reliability, the survey collates established metrics and indicators, following standardized, replicable questionnaire formats [31–33], and reduces respondent fatigue by minimizing time burden. RHoMIS represents a snapshot view of individual households and does not aggregate or integrate information in a causal model based on “average” or “typical” household behavior.

**Table 1:** Lean data indicators collected through the RHoMIS household survey.

Indicator	Description	Unit
Household size	Household members summed up by male adult equivalent (MAE) values, accounting for different caloric energy needs and labor productivity of different gender and age groups	MAE
Household type	Marital status and gender of current household leadership. Options include: Couple, Single woman, Single man, Married woman with permanently absent spouse, Married man with permanently absent spouse	-
Land holdings	Total arable/grazing land owned by the household	Ha
Livestock holdings	Total amount of livestock, including all species, owned by the household	Tropical livestock units (TLU)
Crop diversity	Total number of different crop species cultivated during the past year	-
Livestock diversity	Total number of different livestock species owned at the moment of survey	-
Market orientation	Share of total agricultural production (in kcal) that has been sold during the past year	%
Food Availability	Potential amount of food energy generated by all on- and off-farm activities of the household, including the potential food energy bought from cash income	kcal/ MAE/ day
Number of food insecure months	Number of months the household experienced insufficient access to food of decent quality during the past year	-
Household Dietary Diversity Score (HDDS), Good Season	Number of items out of 12 different food groups (e.g., legumes, vegetables, eggs, etc.) consumed regularly by the household during the recent good season	-
Household Dietary Diversity Score (HDDS), Lean Season	See above, but during the recent lean season	-
Farm income	Total income generated through sale of farm products during the last year	US$/year
Off-farm income	Total income generated through off-farm activities during the last year	US$/year
Greenhouse gas emissions	Total on-farm greenhouse gas emissions	kg CO_2_ equivalents/ year
Women’s decision-making agency	Women’s and female youth’s cumulative share in household decision-making about benefits from on- and off-farm activities	%
Men’s decision-making agency	Men’s and male youth’s cumulative share in household decision-making about benefits from on- and off-farm activities	%

Forty-four villages were randomly selected from administrative village lists for data collection (20 villages each in Region 1 and 2, and 4 villages in Region 3). At each village, 12 farming households were randomly sampled from lists provided by local extension officers. Two teams of four enumerators conducted the survey within a period of two weeks through face-to-face interviews at meeting points in the villages. Data was recorded and digitized on spot using the *Open Data Kit* software [34] on Android smartphones or tablet computers. The survey resulted in a total of 521 successful interviews with household heads.

#### Household performance indicators

Existing applications of the Positive Deviance approach have typically focused on single goals, such as health or nutrition. Our analysis intended to explore successful household behavior in light of possible trade-offs between different goals of current agricultural development paradigms. Despite ongoing debate, widely agreed broad goals include food security, nutrition, income, environmental sustainability, and social equity [35–37]. For each of these goals, we selected one indicator (see Table 2) and calculated household scores from RHoMIS data (see Table 1). Our choice of indicators was limited by data availability and intended to maximize ease of interpretation of the indicators to facilitate our analysis. Future applications may need to include more rigorous stakeholder consultation to select an agreed set of indicators.

**Table 2:** Development goals and household performance indicators used for approximation. Indicator definitions in text.

Goal	Household performance indicator
Food security	Caloric food security
Nutrition	Dietary diversity
Income	Cash income
Environmental sustainability	Greenhouse gas emissions
Social equity	Gender equity

**Caloric food security.** We approached food security by households’ consistent access to sufficient per capita food energy, giving both consistency and sufficiency equal importance. For sufficiency, we estimated household food energy needs by multiplying household size (in male adult equivalents, MAE) by 2,550 Kcal, Tanzania’s official recommended daily calorie intake per MAE [38]. The MAE concept accounts for different energy needs of household members of different genders and ages [33]. We then divided household food availability [39] by the obtained value and capped results at 100%. For consistency, we used the number of food-secure months. We then conducted a principal component analysis on the two measures and used the first loading (which explained 57% of variance) as a composite indicator of household food security.

**Dietary diversity.** Regular consumption of diverse food is crucial to a healthy nutrition. To determine household dietary diversity, we took the harmonic mean of households’ HDDS scores [31] in the good and lean season, respectively (see Table 1). Unlike the arithmetic mean, harmonic mean overemphasizes lower values in the sample, generally leading to lower means. This accounted for our view that the implications to health and well-being through low nutritional diversity in one season cannot be fully balanced by a high diversity score in the other season.

**Cash income.** We defined disposable household cash by the sum of income from farm-gate sales and off-farm activities.

**Greenhouse gas (GHG) emissions.** Environmental sustainability concerns many aspects of farm management (water, soil, biodiversity) that are difficult to cover in a single indicator that would still be easy to interpret. Low farm GHG emissions are not only relevant to global climate change, which is a concern of climate-smart agriculture [3], but are also linked to agricultural practices with local environmental benefits, such as sound soil fertility management, crop rotation, and low use of chemical inputs [40]. To calculate household GHG emissions from practices reported by the households, RHoMIS uses the IPCC Tier 1 approach [32], adding up CO_2_-equivalents from the following emission sources and using standard emission values from literature: livestock enteric fermentation, mineral fertilizer application, manure management, plant residue management, land use area and type, and plant-borne trace gas emissions. Because in our analysis, lower emission values imply higher sustainability, we multiplied resulting emission values by -1, resulting in increasing scores with decreasing emissions.

**Gender equity.** Social equity implies a fair distribution of power and benefits among many social groups, and an important societal contrast in decision-making power and benefit sharing in small-scale agriculture remains between women and men [41,42]. We therefore approach social equity by a gender equity indicator, which covers one important aspect of intra-household social equity. We calculated this proxy from the relative shares of household decision-making undertaken by women and men, respectively (see Table 1). We defined a gender-equitable situation, where decision-making is shared equally between genders, as 0.5. We then discounted deviations from the gender-equitable situation differently by household type (e.g., whether households were woman- or man-headed). The formulae are shown in the Supporting Information (S1 Table).

For each performance indicator, we capped outliers by replacing unrealistic performance scores with the maximum value observed within a realistic range. Outliers were identified by graphical plotting.

#### Defining and calculating deviance

**Fig 2:**
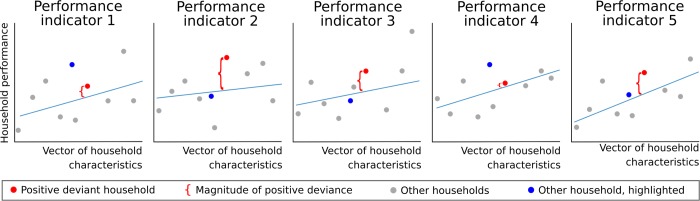
Conceptual figure demonstrating how performance indicators were determined from households’ residuals over performance models. Light blue lines show median regressions, where performance increases with enabling household characteristics (e.g., land endowment). Positive deviants (red) are not the most successful households in absolute terms, but consistently perform better than predicted, unlike other households (see the blue dot).

We were interested in exceptional livelihood performance driven by individual household decisions and behavior. Positive deviance does not mean “a household achieves strong performance”, but rather “a household’s performance is stronger than expected”. Therefore, to identify positive deviants, we transformed absolute performance into relative performance. For each dimension separately, we fit a median regression to data, using multiple household characteristics as explanatory variables to account for external determinants of performance (see below). Each household’s relative performance was thus described by the five resulting regression residuals, quantifying the difference between observed performance and performance expected based on the external determinants. We used these residuals as indicators of relative household performance (Fig 2).

As regression covariates, we used the following household variables: land endowment, livestock endowment, household size, region, and market access, all of which are known to influence livelihood outcomes [37]. Although these variables are not entirely external drivers, as they may also reflect the household’s ability in accumulating assets (land and livestock), they can be seen as constants within the scope of the intervention decisions this method is targeting. To estimate market access, we calculated the mean market orientation (see Table 1) of all households from a same village and used this average observed market utilization as a proxy for potential market access. With intra-household differences within villages evened out, we assumed that market utilization generally reflects potential market access. We eventually selected best fit performance models and included explanatory variables by the Akaike Information Criterion [43].

#### Pareto-optimal household performance

**Fig 3:**
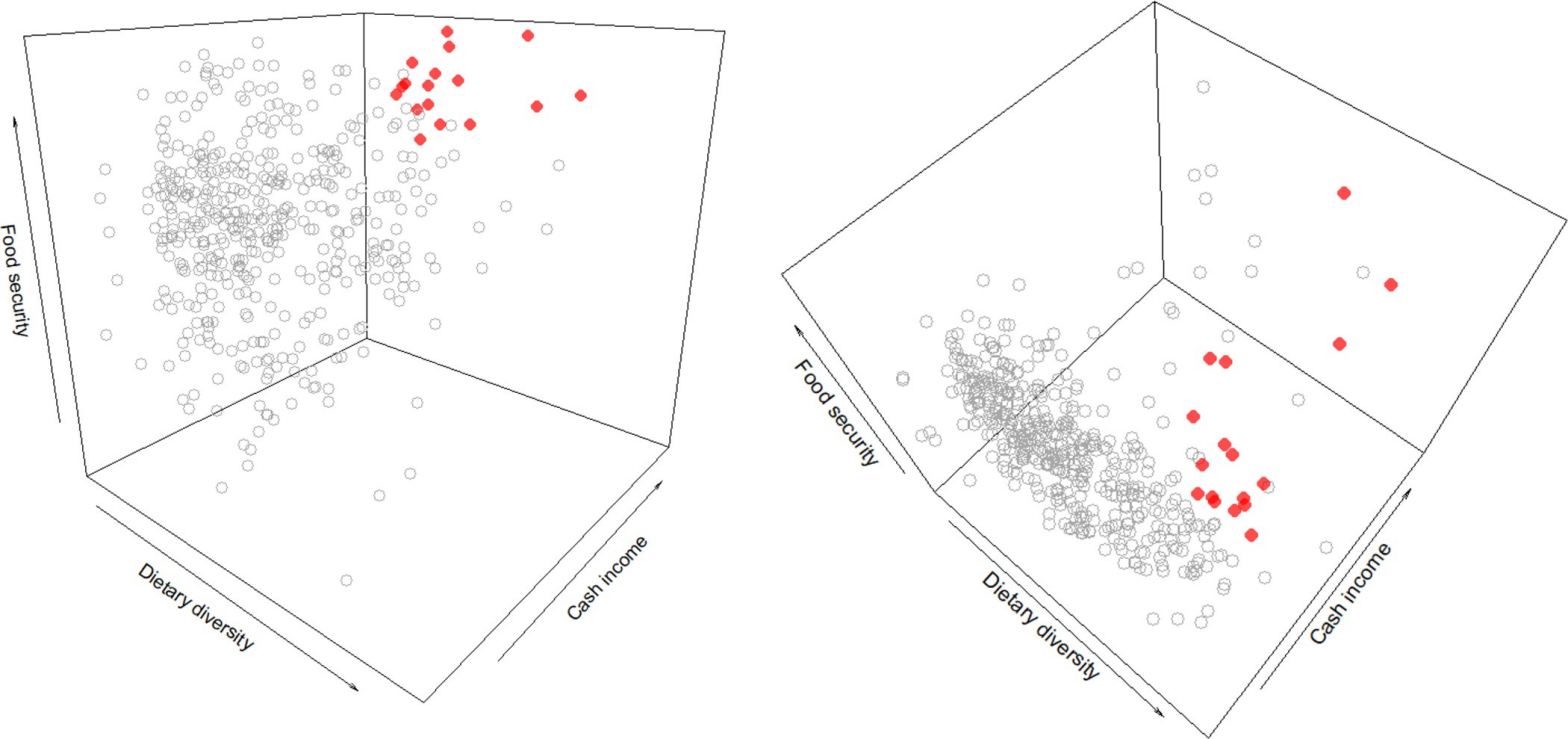
Location of positive deviants and other households in a three-dimensional space of household performance. Positive deviants in red, other households in grey, two perspectives on the same space. In all dimensions individually, some positive deviants are outperformed by other households, but those households suffer stronger performance losses in the respective other two dimensions.

We defined positive deviants as households with Pareto-optimal household performance regarding the five performance indicators. Pareto-optimality does not require that positive deviants perform better than other households in each individual dimension (Figs 2 and 3). Pareto-optimal household performance means positive deviants outperform other households with equivalent characteristics in at least one dimension without being outperformed in any other dimension. This implies they optimize overall outcomes by dealing better with existing trade-offs between performance indicators. We identified positive deviants by searching for Pareto-optimal household performance in a five-dimensional space of performance scores, using the *emoa* package [44] in the R environment [45]. To obtain a reasonable number of positive deviant households in the case of our data, we ran the search twice. After the first search, we excluded the “rank 1” positive deviants from the sample and repeated the search for non-dominated households. We identified a set of “rank 2” positive deviants, which are dominated exclusively by households from the rank 1 Pareto front. In the remainder of this study, “positive deviants” refers to both groups pooled. Given the difficulty of imagining a Pareto front in a five-dimensional space, we here illustrate the concept using three dimensions (Fig 3). To create this figure, we fit a Pareto front to just three performance indicators (dietary diversity, caloric food security, cash income) in our data, and show the position of positive deviants in a three-dimensional space.

The focus on Pareto-optimality embraces diversity and does not privilege any farming style: Households that emphasize caloric food security (e.g. by intensified grain production) can be positive deviants as much as households that emphasize income generation (e.g. by value-adding). But for Pareto-optimality, the individual performance gains must imply smaller losses in the other dimensions compared to other households, which are thus more strongly affected by trade-offs. Positive deviants with diverse priorities and activities will simply lie at different positions of the five-dimensional Pareto-front.

Households engaged in emission-intensive activities, such as cattle fattening or mineral fertilizer use, can also be positive deviants, although we use low GHG emissions as one performance indicator. Firstly, performance models consider livestock holdings, so any household’s performance is always its deviation from the expected emissions level with given livestock holdings. Secondly, a positive deviant may even present high relative GHG emissions, if these do translate into increased performance in the other dimensions (e.g., generating income by value-adding dairy products, or higher crop yields).

#### Quantitative analysis of positive deviance

To inform strategic decision-making on interventions, we determined for which indicator and for which types of household positive deviance was strongest. We compared positive deviance both between the different dimensions of performance and along gradients of resource endowments.

To this end, we first standardized the five distributions of household performance indicators by z-transformation. Within each dimension, we subtracted the distribution mean from each score, then divided through the standard deviation. This quantified all performance scores by their distance from the mean in standard deviations, making the five indicator distributions comparable despite originally different units and scales. We then calculated mean positive deviance of discrete sub-groups of positive deviants. We defined such sub-groups by household resource endowments in land and livestock. By disaggregating effects by these two key productive assets only, we intended to provide intervention agents and development planners with a simple heuristic of positive deviance in the five performance dimensions across diverse resource contexts. For this, we stratified the household sample by deciles of productive land endowments and by the median of livestock endowments (which was close to 0). The resulting 20 resource strata were thus characterized internally by similar land size and, roughly, presence or absence of livestock. We then calculated mean positive deviance of positive deviant households by performance indicator and for each resource stratum. The stratification was also used for the selection of cases for qualitative follow-up research (see next Section). To identify trade-offs between the five dimensions in realizing positive deviant outcomes, we also calculated Pearson’s correlation coefficients between the magnitudes of positive deviance in the individual dimensions.

### Identification of positive deviant practices

#### Selection of households for follow-up inquiry

Our goal was to carry out in-depth qualitative research with a diverse sub-sample of positive deviants. We selected one positive deviant household per resource stratum, applying a stepwise procedure that maximized overall diversity in household characteristics. Two of the 20 resource strata did not include any positive deviant. For the other 18 strata, we always gave preference to rank 1 positive deviants over rank 2, where rank 1 positive deviants existed. We selected the specific subset of 18 positive deviants that had highest overall diversity in terms of household size, land endowments, livestock endowments, and market access. This was the set of 18 households with maximum mean crowding distance [46] regarding those four characteristics (we excluded region, a categorical variable).

#### Interviews and farm visits

Of the 18 households we selected as case studies for more in-depth exploration of livelihood choices, we were able to meet 15 household heads in 12 villages. They were the same persons who had responded the lean data household survey. With every respondent, we first carried out an exploratory, semi-structured interview about the household’s activities (1–3 hours), and then visited at least one farming plot together. We intended to capture all activities related to food production, storage, processing, consumption, income generation, natural resource management, and access to information, paying special attention to any details that seemed unusual (interview guideline in Supporting Information, S1 Text).

The objective of the interviews and farm visits was to identify any practices that were uncommon among most rural households and thus plausible explanations for the positive deviants’ superior performance. During the interviews, we asked follow-up questions about any activities that seemed outstanding at first view. To decide which household practices were indeed uncommon, we relied on three strategies: Firstly, we also interviewed three household heads in the research region who had not participated in the lean data survey. Though we cannot determine whether they would have been positive deviants or not, we treated them as non-positive deviants. Secondly, we relied on our own experience in local farming context (especially author MGM, who participated in all interviews). Thirdly, we asked the positive deviant farmers, who often cited travels, recommendations from friends or extension agents, or personal creativity as inspiration for engaging in uncommon practices. Irrespective of the source of knowledge, we regarded as positive deviant practices all livelihood-related practices that were both uncommon in the research region and established beyond experimental stage at the positive deviant household. In joint deliberations, the authors who carried out the interviews (JS and MGM) analyzed interview notes to decide which household activities fulfilled these criteria, leading to an agreed list of observed positive deviant practices.

### Positive deviants as models for similar households

In prioritizing development options for target households, we intended to account for household diversity by suggesting multiple intervention options according to individual household characteristics. We tried to avoid both over-targeting of practices (closed to households’ diverse preferences) and under-targeting (letting all households choose from the full set of options). To provide a useful heuristic tool to development agents, we here focused, for each target household, on the practices found with the three positive deviants that were most similar to it. We suggest this limited number of positive deviants, along with the set of practices found with them, should inform focused discussions about viable, individually suitable development narratives grounded in local reality, through “case-based reasoning” [47].

We approached similarity between target households and positive deviants by their household endowments in six key resources: agro-ecological ability, labor, financial capital, land holdings, livestock holdings, and social capital (proxy definitions, based on RHoMIS indicators, in Supporting Information, S2 Table). For each household included in the baseline survey, we identified the three most similar positive deviants from the sub-sample we had visited (see previous section) by calculating Euclidean distance on the six resource levels. We defined for each of these target households the three positive deviants with lowest Euclidean distances (its 1^st^, 2^nd^, and 3^rd^ “resource homologues”). Euclidean distance treats positive and negative deviations (whether the household’s resource levels were higher or lower than those of the positive deviant) equally, accounting for some fluidity and compensation effects between resources (e.g., livestock and capital are often mutually convertible to certain extent).

### Ethics statement

This study conforms with the principles of the 1964 WMA declaration of Helsinki. Approval for survey data collection was obtained from both project leadership at Bioversity International and the directorate of Naliendele Agricultural Research Institute. Research permissions for the RHoMIS survey and positive deviant interviews were also obtained from District Agricultural, Irrigation and Cooperative Officers (DAICOs) in all administrative districts included, conforming with the requirements of the Tanzania Commission for Science and Technology (COSTECH). The ethics committee at the Faculty of Life Sciences at Humboldt University Berlin was not involved because its guidelines do not require prior ethical approval for a household survey like this. Survey participants were not particularly vulnerable, data was processed in anonymized form, and survey participants had the possibility to skip questions. Explicit oral informed consent was obtained from all survey participants prior to survey enumeration and documented as opening question in the RHoMIS survey. If consent was denied, enumeration stopped after one question. Permission for obtaining oral rather than written consent from survey respondents was granted by DAICOs, given literacy limitations among the target population.

## Results

### Characteristics of positive deviants

Out of the 521 surveyed households, 54 were positive deviants, achieving rank 1 (n = 12) or rank 2 (n = 42) Pareto-optimal performance for five dimensions of household performance. Positive deviants stood out due to their strong *relative* performance considering their specific household characteristics. Nonetheless, for three dimensions (caloric food security, dietary diversity, and cash income), positive deviants on average also achieved higher absolute performance than other households. Overall, they did not realize higher gender equity than other households, and even showed slightly worse indicator values for GHG emissions in absolute terms (Table 3).

**Table 3:** Selected socio-economic characteristics and median performance scores of surveyed households.

	Positive deviants	Other households
Number of households	54	476
In region 1 / 2 / 3	**59% / 26% / 15%**	**43% / 48% / 9%**
Woman-headed households	30%	29%
Mean age of household leader	44.4	47.9
Education of household leader:		
Illiterate / Literate / Primary / Secondary	**2% / 4% / 76% / 19%**	**8% / 7% / 80% / 5%**
Marital status: Married	91%	86%
Mean household size (MAE)	4.34	4.21
Mean land endowment (Ha)	4.09	3.89
Mean livestock holdings (TLU)	0.28	0.36
Mean livestock diversity	**1.06**	**0.79**
Mean crop diversity	4.26	3.96
Presence of off-farm income	43%	30%
Median caloric food security (unitless)	**0.67**	**0.23**
Median dietary diversity (food groups)	**6.56**	**4.00**
Median cash income (US$/year)	686	281
Median GHG emissions (CO_2_-eq/year)	**395**	**212**
Median gender equity (%)	0.33	0.33

Significant differences (p < .05) in household characteristics are shown bold (Student’s t-test / Pearson’s Chi square test).

Positive deviants did not differ from other households with respect to gender ratio, age, marital status, household size, land endowment, and livestock endowment (Table 3). Positive deviants had, however, achieved higher levels of formal education. They were also not evenly distributed across regions, with significantly fewer positive deviants in Region 2 than in the other two regions. Both positive deviants and other households had relatively low mean livestock endowments. Mean livestock diversity, however, was higher for positive deviants than for other households.

### Overall patterns in positive deviance

Overall, mean positive deviance was strongest for caloric food security, followed by GHG emissions and cash income (Table 4, last row). For gender equity, positive deviants on average actually performed slightly weaker than expected (Table 4, last but one row). Individual positive deviants achieved diverse outcomes regarding the specific magnitudes of positive deviance in each dimension (see the examples in Table 5), and there were both weak positive and weak negative correlations between these magnitudes (Table 6).

**Table 4:** Mean deviance by performance dimension and aggregated resource strata.

	Caloric food security^a^	Cash income (US$/a)	Dietary diversity (food groups)	Gender equity (%)	GHG emissions (CO_2_-eq/a)^b^	n
Land size strata						
1+2	0.79	986	2.6	1	379	13
3+4	0.56	251	1.4	2	722	9
5+6	0.83	592	1.6	-9	834	7
7+8	0.60	461	1.9	1	359	13
9+10	0.70	3140	2.7	-5	479	10
Low livestock	1.01	1251	3.4	-5	-285	15
High livestock	0.56	994	1.6	-2	813	39
Overall mean	0.69	1066	2.1	-1	508	54
*Overall mean (scaled*, *unitless)* ^c^	*0*.*65*	*0*.*07*	*-0*.*93*	*-1*.*52*	*0*.*33*	54

^a^ Caloric food security scores are products of a principal component analysis and unitless.

^b^ Values refer to reductions against expected values, so high values are desirable.

^c^ To allow comparison of deviance across dimensions of performance, means were also scaled by z-transformation (last row). For each dimension, the unitless value quantifies mean deviance by the difference from the population mean in standard deviations.

**Table 5:** Deviance of individual positive deviants that were visited for qualitative follow-up research, practices identified with them, and numbers of resource homologue households per positive deviant.

Positive deviant (inter-viewed)	Magnitude of deviance					Practices^b^	Number of resource homologue households^c^			
	Caloric Food security (unitless)	Cash income (US$/a)	Dietary diversity (food groups)	Gender equity (%)	GHG emissions (CO_2_-eq/a)^a^		1^st^	2^nd^	3^rd^	*Total*
I	0.74	202	1.35	14	491	Sc	253	53	41	*347*
II	1.35	826	0.28	4	812	Ic	1	53	23	*77*
III	0.10	698	-1.71	1	4,492	Mb, Pi, Sc	5	7	56	*68*
IV	0.62	539	4.07	0	-161	Sc	8	10	3	*21*
V	0.29	127	3.38	1	1,618	Lb, Mt, Ss	31	56	2	*89*
VI	0.56	331	3.72	0	-103	Lb, Sc, Wl	5	7	6	*18*
VII	0.01	113	0.30	0	2,585	Pu, Tn, Sc	4	31	61	*96*
VIII	1.60	129	-0.13	1	662	Wl	59	267	0	*326*
IX^d^	1.70	10,477	2.00	1	2,338	-	-	-	-	*-*
X	1.35	2,081	2.73	1	-539	Lb, Mt, Pu, Wl, Tb	54	0	1	*55*
XI	1.49	1,819	3.61	4	-633	Cs	9	7	6	*22*
XII	0.00	649	4.34	2	676	Cs, Sp	25	21	8	*54*
XIII	1.12	513	2.56	-4	-349	Sc, Ss, Wl	52	7	289	*348*
XIV	1.54	964	3.57	1	-274	Cp, Mt	15	2	25	*42*

^a^ Values refer to reductions against expected values, so high values are desirable.

^b^ See Table 6

^c^ As most (1^st^), second-most (2^nd^) and third-most homologue (3^rd^)

^d^ No deviant practice identified

**Table 6:** Pearson’s correlation coefficients between dimension-specific magnitudes of positive deviance.

	Caloric food security	Cash income	Dietary diversity	Gender equity	GHG emissions
GHG emissions	-0.22	0.24	-0.18	-0.16	1
Gender equity	**-0.29**	**-0.35**	-0.19	1	
Dietary diversity	0.26	0.20	1		
Cash income	**0.32**	1			
Caloric food security	1				

Significant relationships (*p* < .05) are shown bold.

Both land and livestock endowments seemed to influence average positive deviance (Table 4). For the smallest and largest farm sizes, positive deviance was strongest for cash income and dietary diversity. For GHG emissions, however, medium-sized farms showed strongest deviance. Household with low livestock endowments had, on average, stronger positive deviance for caloric food security, cash income, and dietary diversity. In turn, households with higher livestock endowments performed more strongly for gender equity and GHG emissions.

### Positive deviant practices

**Fig 4:**
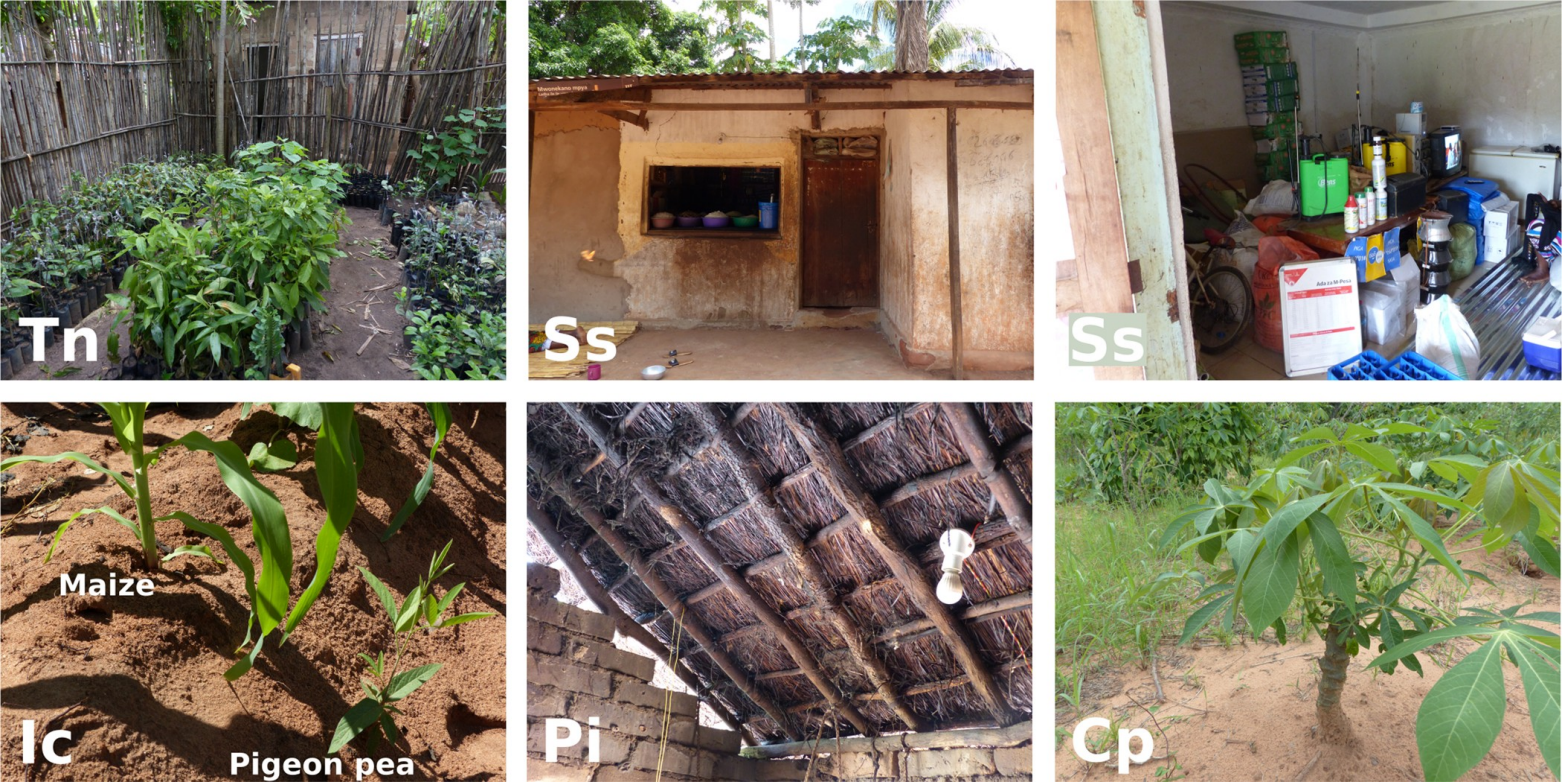
Examples of deviant practices observed with positive deviants. Tn, tree nursery; Ss, small shop; Ic, resource-efficient intercropping of maize and pigeon pea; Pi, poultry intensification; Cp, production of cassava planting material.

Through interviews and farm observations with a subset of 15 positive deviants, we identified 14 “positive deviant” practices (Table 7 and Fig 4). We found seven of these practices with single positive deviants only, but other practices were applied by up to six positive deviants. At one household, we did not identify any uncommon practice. Other positive deviant households were, on average, engaged in 2.2 of the practices, simultaneously (maximum: 5).

**Table 7:** Positive deviant practices observed with positive deviant households and total numbers of households that would be targeted with each practice, following the resource homologue approach (n_max_ = 521).

Practice	Code	Mechanism	Frequency observed	Number of target households	% of total
Production of cassava planting material	Cp	Generating income by producing and selling quality cutlings of an improved cassava variety	1	42	8
Investments into improved crop storage	Cs	Decreasing post-harvest losses by investing into improved crop storage constructions or triple layer PICS sacks [48]	2	76	15
Resource-efficient intercropping of maize and pigeon-pea	Ic	Decreasing plant competition for environmental resources by sowing pigeon pea at the lower end of the shadow-side slope of ridges	1	77	15
“Livestock bank”	Lb	Increasing household resilience by maintaining ruminant livestock even against short-term utility logic, for sale in emergency situations	3	107	21
Milk business	Mb	Generating income by pooling small-scale cow milk production with neighbors and sending bulk produce to buyer in town via public transport	1	68	13
Shared use of mechanical tillage	Mt	Increasing economic farm efficiency by pooling capital with neighbors to hire a tractor-tillage service provider, saving wages for manual tillage laborers	3	131	25
Intensified poultry production by artificial lighting	Pi	Increasing poultry production per unit of time by investing into a solar power-driven light bulb, enforcing artificial lighting all night and increasing daily food intake of poultry	1	68	13
Up-scaled poultry production	Pu	Increasing production and productivity of poultry by investing into bigger, more secure coops and/or new animals of improved breeds	2	96	18
Meticulous scheduling of labor allocation during land preparation and sowing of crops	Sc	Decreasing risk of crop failure by applying agronomic knowledge and skills in proper priority-setting for time and labor allocation during early phases of the growing season	6	521	100
Speculative purchase and stockpiling of crop	Sp	Generating income by investing into buying crop when prices are low, renting storage space, and selling when prices are high	1	54	10
Small shop for ago-inputs, and building materials	Ss	Generating income by running a small village shop, often employing family members, selling agro-inputs sometimes on a commission base	1	348	67
Transportation business	Tb	Generating income by investing into a van that connects two urban centers multiple times per day, with a family member employed as driver	1	55	11
Commercial tree nursery	Tn	Generating income by producing and selling tree seedlings, including grafted cashew seedlings	1	96	18
Wage labor	Wl	Generating income by dedicating labor to off-farm wage work	4	421	81

### Resource homologues

For each household, three positive deviants were identified according to their relative similarity to the household in resource endowments (“resource homologues”). For 323 households (62%), the homologues were, in varying orders, positive deviants I, VIII, and XIII (see Table 5). For these households, priority interventions might emphasize farm labor scheduling (Sc) and off-farm income generation through a small shop (Ss) or wage labor (Wl). The shares of households associated to each individual practice by the resource homologue approach ranged from 8% for the production of cassava planting material, to 100% for farm labor scheduling (Table 7).

## Discussion

### Diverse positive deviants may inform household-specific intervention choices for heterogeneous target households

We designed and tested a method to identify farming households that achieve unexpectedly strong performance (positive deviants) and identified diverse practices that may have contributed to their superior outcomes. Positive deviants, about 10% of the survey sample, represented the overall household diversity well, including, e.g., very small and very large farm sizes. Uncommon practices were found even among the least wealthy households, implying that positive deviants indeed made superior household decisions, instead of just overstating performance in the baseline survey. Regional imbalance in the distribution of positive deviants may be due to different intensities of trade-offs at different locations, e.g. due to distinct dominating farming systems. The higher livestock diversity that was observed with positive deviants might in itself represent a positive deviant practice, since livestock diversification is associated with multiple livelihood indicators [49]. That positive deviants on the whole have received higher levels of formal education is not surprising, as education is known to drive on-farm innovation processes, especially by reducing risk aversion [50], and may give farmers more lucrative off-farm labor opportunities.

The diversity in resource context among positive deviants suggests that household performance heterogeneity is at least partly due to individual decisions and behaviors. It also implies that for most households, positive deviants in relatable household context (with similar productive resources, location, farming system) may exist. This heterogeneity of success cases could be exploited to accelerate local development: For any household, the resource homologue approach identifies positive deviants as most similar solution templates, which may serve as starting points for empirically grounded discussions around adaptations in farm decision-making. This provides development agents with a heuristic for household-specific prioritization of intervention options, rather than assigning households to broad clusters, which may mask important parts of heterogeneity [51]. Since the group of positive deviants was highly diverse, such discussions may take the heterogeneity of target households into account. Given the empirical nature of insights from our method, kickstarting practitioners’ discussions about interventions may require less assumptions than alternative methods that assess the effects of new practices based on household data [8]. This empirical focus, however, restricts analysis to practices that are already in use in the study area, meaning that some promising technology options, as well as institutional change, may be left out of discussions.

### Identifying locally viable practices for agricultural development does not require complex econometric or system modeling

Studying the identified household success cases should allow development agents to draw plausible links between unique practices and performance outcomes. This does not require a comprehensive inventory of household activities, data-intensive system modelling or more complex econometric analysis. The method can be used by development agencies, such as NGOs or extension services, to rapidly identify a list of candidate practices that can then feed into empirically grounded discussions on intervention priorities. While the first, quantitative step requires knowledge on data cleaning and statistical analyses, it can be carried out by remote collaborators, e.g., researchers. For the second, qualitative step, the focus on empirical success cases instead of causalities, data means, and trends likely makes it easier for stakeholders not familiar with quantitative methods to participate meaningfully in discussions about viable development strategies.

Interestingly, the 14 positive deviant practices identified in this study differed from what has previously been suggested as “best-bet” solutions in similar context, such as rainwater harvesting, or biochar utilization [51]. Visiting more positive deviants and repeating the inquiry at another time of the year likely would have led to more practices, and possibly a larger overlap with the practices presented in the literature. Including a different number of households in the quantitative survey might have led to different sets of positive deviants and associated practices. The same is true for alternative indicator definitions, as we used available data from the RHoMIS survey, which provides a rapid, but also necessarily limited view of household performance. Defining performance indicators differently would likely have identified a different set of positive deviants, possibly with different practices.

More importantly, however, we identified concrete local realizations of certain practices (e.g., “resource-efficient maize-pigeon pea intercropping”), while many prioritization exercises describe broad collections of practices (e.g., “intercropping” without specifying the crops) [20,52]. The concrete practices we identified may be more directly applicable for other households. Promoting these directly observable cases may inspire others to test these practices on their own farms [53]. This can lead to further formal and informal adaptation and experimentation, perhaps supported by systematic on-farm experimentation formats [54,55].

In suggesting interventions, development agents should mind some important limitations to the effects that the identified practices can have on household performance. For example, finite societal demand for some of the produced goods and services (e.g., tree seedlings, village shops) may cap the total numbers of adopting households that may sustainably improve their livelihoods. As expected, practices that likely involve market competition (Cp, Sp, Ss, Tb, and Tn) in general seem less widely applicable than other practices, following the resource homologue approach. Potential negative societal externalities of some of the identified practices also deserve attention. For example, speculative stockpiling of crop after harvest may increase consumer prices and aggravate food insecurity of landless people. Likewise, replacing manual tillage by renting a tractor can reduce income opportunities for low-skilled, often landless rural people.

### Performance differences between positive deviants and other households suggest locally promising intervention domains

Positive deviants demonstrate that household performance can be improved in each of the five dimensions. Nonetheless, there are important differences that may inform decision-making on interventions and research. For example, positive deviants on average performed considerably better than other households regarding caloric food security, but positive deviance was relatively weak for gender equity. Those households that stood out particularly for their gender equity tended to have below-average positive deviance for the other dimensions, and vice versa. While there seems to be strong potential for interventions that target food security, this trade-off indicated that less opportunities exist for improvements in gender equity without affecting other indicators negatively.

The difference may, however, also reflect current priorities of households (more experimentation around production than around social relationships) or mean that progress in gender equity requires more radical innovation, which may be less likely to develop through farmers’ own experimentation [56]. Follow-up research could explore possible solutions. But future applications of the Positive Deviance approach might also reach different conclusions by using more comprehensive conceptualizations of gender equity, as we used a relatively narrow perspective on intra-household responsibilities. In addition to partial conceptualizations, our choice of household performance criteria, which was based on current development paradigms, may risk identifying success cases that are not preferred by local stakeholders. More participatory agenda-setting could be used to increase impacts in future uses of our method.

Positive Deviance constitutes a distinct, complementary approach to other participatory approaches in agricultural research. Other qualitative research approaches are also able to generate concrete example cases [57], but our method is unique in applying a highly systematic procedure with objective criteria to select a diverse subset of well-performing households. A step-wise research procedure of inquiry enhances the reliability and replicability of our method: Although the use of farmer self-reported quantitative data can introduce new forms of bias [58], the subsequent qualitative research step filters out low-quality data, as the farm visits allowed us to distinguish actual positive deviants from households that might have over-reported performance. Also, sampling diverse example cases from a reasonably large group of positive deviants (~10% of all households) helped to avoid a narrow focus on the most extreme outliers, which may suffer more from low data quality (due to exaggeration or data entry mistakes). This principled approach likely reduced certain types of bias reported in participatory research due to less systematic selection of households and data processing [16,59]. Compared to other participatory approaches, however, our method requires an investment into prior survey data collection. Even so, in projects that require quantitative impact assessment, the RHoMIS survey can serve both as baseline and as input to the analysis of Positive Deviance.

## Conclusions

We designed a new method for informed planning of household-level smallholder agricultural development interventions by operationalizing the Positive Deviance approach. A novelty in our application of the approach is the simultaneous focus on multiple objectives in agricultural development, based on the concept of Pareto-optimality. We explored how cases of surprisingly strong multi-objective household performance (positive deviants) can be identified from survey data, and how the diversity in the dataset can be exploited to inform the household-specific prioritization of intervention options for heterogeneous target households. Our analysis explored the differences between positive deviants and other households, generating a list of household-level development options that were proven to work in local context. This type of empirical insights provides valuable inputs to discussions by development practitioners and farmers for planning development interventions that are well-grounded in local context as well as conscious of trade-offs between multiple objectives. In the future, our method may be extended to other use contexts (beyond agriculture) that imply trade-offs between different development goals.

## Supporting information

S1 Table Household-type specific formulae for calculation of a gender equity indicator from RHoMIS data. (DOCX)

S2 Table Calculation of household endowments in six resources that may drive different adoption potentials for novel practices and technologies. (DOCX)

S1 Text Positive deviant interview guideline. (DOCX)

S1 Dataset RHoMIS survey data of 521 rural households in Tanzania. Households and locations have been anonymized. Units can be found in Table 1.
(XLSX)
