# Supplementary material for: Prioritizing options for multi-objective agricultural development through the Positive Deviance approach
Source: PLoS One. 2019 Feb 25;14(2):e0212926. doi: 10.1371/journal.pone.0212926 (PMC6388925; doi:10.1371/journal.pone.0212926)
Supplement: S1 Table — (DOCX) [file pone.0212926.s001.docx]

**S1 Table. Household-type specific formulae for calculation of a gender equity indicator from RHoMIS data.**

| **Household type** | **Formula** | **Comments** |
| --- | --- | --- |
| “Couple” | Women’s decision-making agency (WDA)^a^ | In a couple, WFR = 0.5 implies women and men share decision-making on benefits in an equitable way. |
| “Single woman” | 0.5 – (1 – WDA) | In a single woman household, we define a gender-equitable situation as WDA = 1. Any deviation from 1 means that decision-making was transferred from women to men, leading to a decrease in gender equity. Since we standardise gender-equitable situations as 0.5, we subtract the degree of transferred decision-making from 0.5. |
| "Single man” | 0.5 + WDA | In a single man household, we accept WDA = 0 as a “gender-equitable” situation. This also means that any household responsibilities transferred by single men to women increased gender equity. |
| “Woman –  man lives away” | 0.5 + x * (WDA – MDA^b^)  where  WDA > MDA → x = 0.5  WDA < MDA → x = 2 | We expect the distribution of decision-making power in these households to be an intermediate case between “Couple” and “Single woman”. Like in the case of a couple, WDA = 0.5 implies a gender-equitable situation. However, deviations are discounted, given the expected dynamics of a spatially divided couple: With men living off-farm, we generally expect transferal of decision-making from men to women. As a heuristic, we thus attribute only half weight to such “expected” transferal, leading to lower increases in gender equity. At the same time, responsibilities counterintuitively transferred from women to men are doubled, and lead to a stronger decrease in the household’s gender equity indicator. |
| “Man –  woman lives away” | 0.5 + x * (WDA – MDA)  where  WDA > MDA → x = 2  WDA < MDA → x = 0.5 | See above. In the case of women living off-farm, we give less weight to the expected transferal of responsibilities from women to men, but attribute double weight to transferal of responsibilities from men to women. |
| ^a^ see Table 1  ^b^ MDA = Men’s decision-making agency, see Table 1 | | |
