# Supplementary material for: Prioritizing options for multi-objective agricultural development through the Positive Deviance approach
Source: PLoS One. 2019 Feb 25;14(2):e0212926. doi: 10.1371/journal.pone.0212926 (PMC6388925; doi:10.1371/journal.pone.0212926)
Supplement: S2 Table — (DOCX) [file pone.0212926.s002.docx]

**S2 Table. Calculation of household endowments in six resources that may drive different adoption potentials for novel practices and technologies**

| **Household resource** | **Data inputs from RHoMIS** | **Calculation** |
| --- | --- | --- |
| Agro-ecological ability | A) Education level  Formal education level of household head (illiterate = 0, literate = 0.5, primary level = 1, secondary level = 2)  B) Farmer-to-farmer information exchange  Number of specific agricultural topics the household has previously received information about from other farmers (e.g., “pests”). Integer scale (0-14).  C) Crop productivity  Defined as Calories produced per Ha cultivated. | First loading of a principal component analysis on A, B, and C. |
| Labour | Household size (in MAE),  Land holdings (Ha) | HH size / Land holdings  (With constant technology, the person / land ratio is a good predictor of the availability of labour in the household) |
| Financial capital | Total income,  Household size (in MAE) | Total income / HH size  (Per capita income, as this likely defines the availability of surplus for investments) |
| Land holdings | Land holdings (ha) | - |
| Livestock holdings | Livestock holdings (TLU) | - |
| Social capital | A) Group membership  0 if household is not member in any group,  1 if household is member in at least one group (e.g., rural marketing cooperative)  B) Aid / Gift receival  0 if the household has not received any type of public aid or gift in the last year,  1 if yes | First loading of a principal component analysis on A and B. |
