# Supplementary material for: Prioritizing options for multi-objective agricultural development through the Positive Deviance approach
Source: PLoS One. 2019 Feb 25;14(2):e0212926. doi: 10.1371/journal.pone.0212926 (PMC6388925; doi:10.1371/journal.pone.0212926)
Supplement: S1 Text — (DOCX) [file pone.0212926.s003.docx]

**S1 Text. Positive deviant interview guideline**

The following interview questions intend to roughly guide a semi-structured conversation. The interview is preceded by mutual presentation, explanation of the research purpose, and relationship-building through friendly chatting. Both questions and answers were translated between English and Kiswahili on spot.

**CROPPING**

**Crop choice**

Which crops do you grow? How do you decide which crops to plant? What does your choice of crops depend on? Why don’t you grow other crops? Which other crops have you grown throughout your life? Why have you changed your choice of crops?

**Varieties**

Which varieties do you use? Why? Do you change the varieties? Why / why not? Which other varieties do you know? Have you changed your use of varieties over the years? Do your neighbors use the same varieties?

Where does your seed come from? Would you also plant seed from other sources? Have you multiplied you own seed? For which crops?

**Agronomy**

Who showed you how to cultivate the crops? How do you know how to do it? In the household, who knows best how to cultivate each crop? Are there also other ways to grow the crops? Have you tried other ways before? Why do you do it this way and not another way? Would you do it differently if your farm was at a different location?

What do you do when there is a problem you don’t know how to solve? Are there crops you find more difficult to cultivate than others? Why? Which crop is the easiest to grow?

In terms of cropping, what was a major lesson you have learnt throughout the years? What have you discovered that other farmers may not do or know? What practices have you changed along the years? What practice would you still like to learn or understand better?

**Input use**

Which inputs do you use? Why? On which crops? There are also other inputs, why don’t you use those? Which other types of inputs do you know? How do you know which input to use and which amount? Have you also tried different kinds of inputs?

**LIVESTOCK HUSBANDRY**

**Choice of species / breeds**

Which animals do you keep? Why do you keep them (for what purpose)? Why do you keep these species and not others? Have you had other types of animals before? Why have you abandoned those? Do you own a stable number of animals throughout the year?

Do you know the breeds you have? Why do you use these breeds and not others? What would happen if you changed the breed? Where did you obtain the animals you have? Or their ancestors?

**Livestock husbandry practice**

Where do you keep your animals? Why? What do they eat? How does that change throughout the year? Are they kept in the same way all year long?

In the household, who knows best about how to treat the animals? Who takes care of them? Why? Do animals get sick sometimes? What do you do when an animal gets sick? What do you do to prevent them from getting sick? What other measures do you know about? Why do you not apply them on your farm?

**STORAGE AND PROCESSING**

**Harvest storage**

After harvest, what happens to the crop? Where do you store it? Do you treat it before storing? Which problems have occurred to you in relation to crop storage? And to which crops specifically? What was your reaction? Have you changed anything about your storage techniques?

Do you also know other ways of storing crops? Which techniques have you tried? Why? Why not?

**Processing crops** *Flours, juice, ice cream, dried fruit, shelled nuts, roasted nuts, …*

Do you process any crops before storing or selling them? Which crop products do you produce? Why do you do that? If not, which crop products do you know how to produce? Is it difficult to process the crops? Have you processed crops differently in the past? Is there a product that you would like to know how to produce?

In your household, who processes the crops? Who knows best how to do it? Over time, what have you learnt about these processing activities?

**Animal products** *Cheese, milk, meat, eggs, dried meat, feathers, skins, bones, …*

Which animal products does your farm produce? Why do you produce them? Have you tried producing other products? Why did you stop? What animal products would you like to produce in the future?

**COMMERCIALIZATION**

**Market functioning**

Which products from your farm are sold? Where are they sold? How does this happen? Which member of the household is in charge of these activities (*distinguish between different crops, animals, etc.)?* Who decides the prices?

Who decides what is sold, and how much? Why do you sell this and not that? Have you tried selling different products before than the products which you sell now? Do you think that the importance of selling products has changed over the last years? Was that good or bad for you?

Are you satisfied with the prices you get? What can you do to increase the price? What have you tried in the past to increase the money you get for your products? What would be your main advice about selling farm products? What have you learnt over the years?

**OFF-FARM ACTIVITIES**

Does any member of the household ever work outside the farm? Why? How have you found out about this opportunity? Is that a regular arrangement, or just occasional? Was it difficult to find labor? Have you always worked off-farm? Since when does the household do it? Why did you start?

Don’t you need the working hands on the farm? What would happen if there were no off-farm labor opportunities? Would you like to engage more in off-farm work? Why? Why not? If yes, why can’t you do it?

**NUTRITION**

For you, what is important for a healthy nutrition? How do you make sure your household gets a healthy nutrition? In the household, who is responsible for taking care of a good nutrition?

How does your consideration for nutrition influence your farming decisions? Do children need a different nutrition than adults? How does this influence your farming?

*If there is a homegarden:* Why do you maintain a homegarden? What do you cultivate here? What was here before the homegarden? Is it worth the work to maintain a homegarden? Why? Why don’t other people have homegardens?

**HIRING LABOUR**

Do you ever hire people to work for you? For which crops and at which occasions? Why? Why not? With which money do you pay them? Who are the people you hire? Why do they have the time to work on your farm? How do you find them? In the household, who is responsible for hiring workers?

What would happen if no manpower was available? Are there also machines that could perform this work?

**FARM PLANNING, GENDER + DECISION-MAKING**

**Projection of the future**

How will your household and farm change in the next five years? Why do you think this? How can you influence this process? Which plans are you going to realize? Why?

**Decisions**

Have you invested money to make improvements in your farm? Have you ever thought about taking a loan to be able to invest (*for example, for mechanical tillage*)? Why? Why not?

What was the hardest choice you had to take on your farm? Why did you decide the way you did? Why was it a hard choice? What would have happened if you had decided differently? Who else was involved in the decision-making? Who else is generally involved in important decisions that concern the farm and household?

**AGRICULTURAL KNOWLEDGE AND INFORMATION**

**Agro-ecological knowledge**

How do you know when the rainy season will start? What kinds of soils do you have? Compared to your neighbors, are they better or worse soils?

How has the weather changed over the last years? How has this influenced your decisions on the farm?

**Agro-information**

Which information do you receive about farming *(e.g. weather forecasts, market information)*? Why? How do you receive it, and from which source? Is it reliable? Do you check it with other sources? Do your neighbors use the same information? Why? Why not?

How do you know which inputs to use? How do you learn about new products? Do you trust this information source?

What was the last time you made a significant change on your farm? And what was the last time you tried out something new on your farm (*a new crop, a new variety, etc.*)? How did that go? How do you usually proceed when you try out something new?

**ATTITUDES TOWARDS THE ENVIRONMENT**

**Eco-attitudes**

How important is it to protect the environment? Which parts of your farm and household have an influence onto the environment? Which parts of your farm and household have a positive influence? And which parts of your farm and household have a negative influence onto the environment? Why do you find this negative? Have you done anything to change this influence?

What can farmers do to change their influence on the environment? Why would they do that? Who does this already? Why doesn’t everybody do it?
